# Supplementary material for: Identification and Functional Analysis of Tomato TPR Gene Family
Source: Int J Mol Sci. 2021 Jan 13;22(2):758. doi: 10.3390/ijms22020758 (PMC7828616; doi:10.3390/ijms22020758)
Supplement: Supplementary file 1 [file ijms-22-00758-s001.pdf]

Table S1. Primers used for qRT-PCR

Primer name	Upstream primer（5′-3′）	Downstream primer（5′-3′）
SlTPR2	GATTTTTGCCGCCAGGGAAG	GGATCTGCACCCTGTTCCAA
SlTPR4	TGCCAGTTTAGCACCAGGAG	GTTGACCGTTGGTTGAGCAC
SlTPR10	CGTTGCATTTGCAGGTGGAG	ATGTCAACAAGGGAGGGCAG
SlTPR12	GATGCCAGAGGAGGACGATG	AGACACTTCCATTGGCTCCG
SlTPR14	ATACGAGGATGCCTTGGCTG	TCCAGTTCTTCAGTGCGCTT
SlTPR20	TCCTTTGTAGCAAGGCAGGG	GGCTCTCTGCCACTGTTTCT
Actin	TGTCCTATTTACGAGGGTTAT<br>GC	CAGTTAAATCACGACCAGCAAG<br>AT

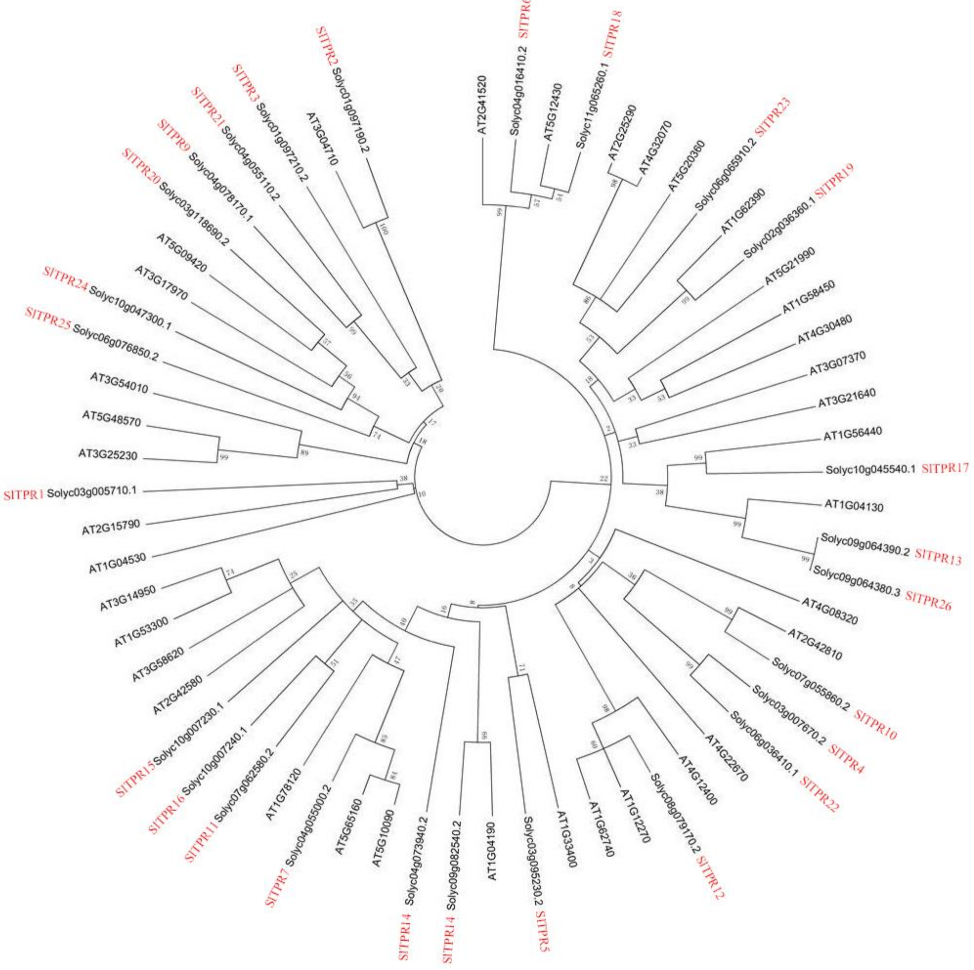

Figure S1. Phylogenetic relationships between tomato and *Arabidopsis TPR* genes

Nine pairs of vertical homologous gene pairs refer to the homologous gene pairs of different species and seven pairs of parallel homologous gene refer to homologous genes in the same species.

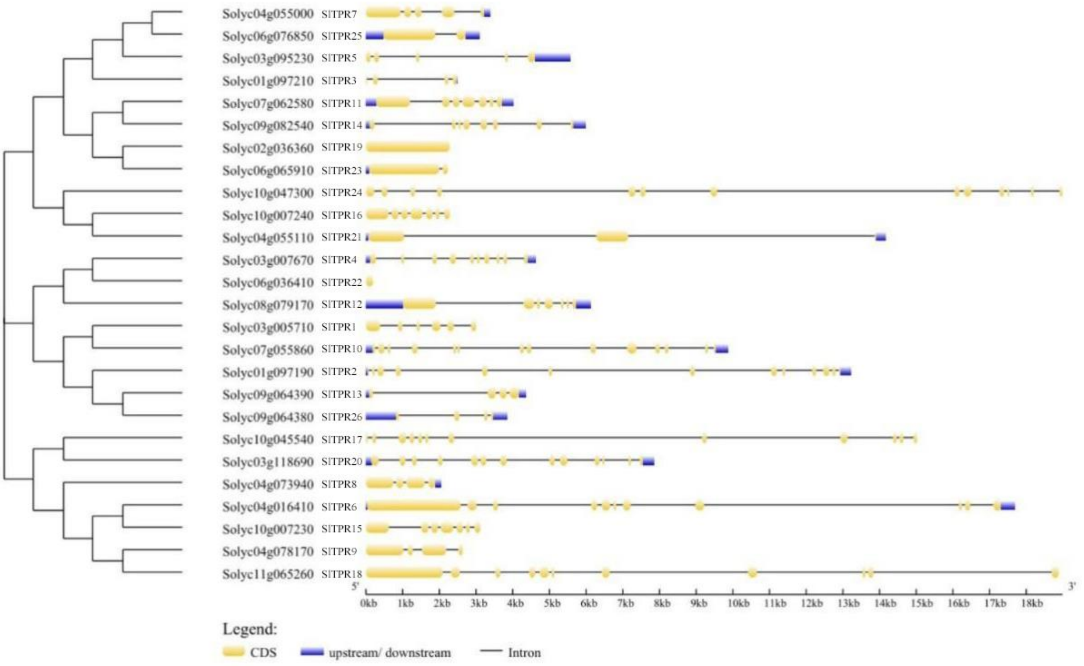

Figure S2. Exon-intron structure of tomato *TPR* genes

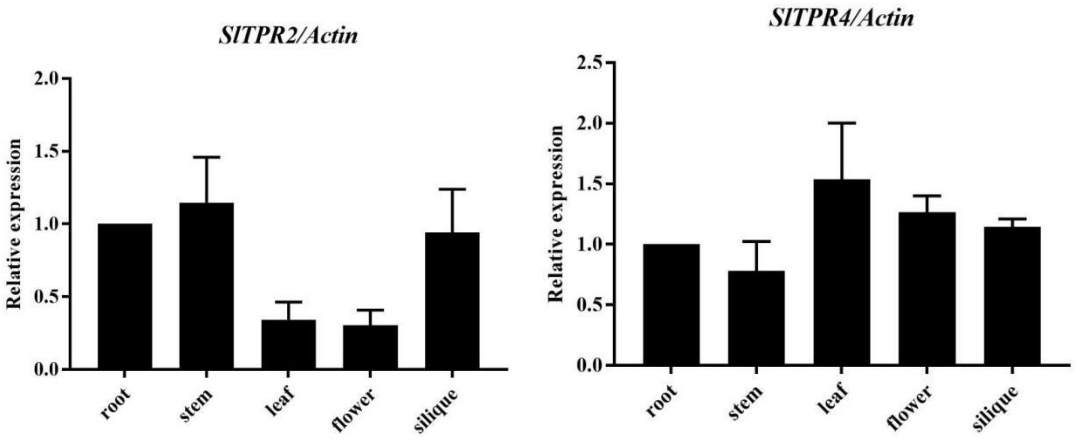

Figure S3. Organ-specific expression of *SlTPR2* and *SlTPR4* gene in tomato

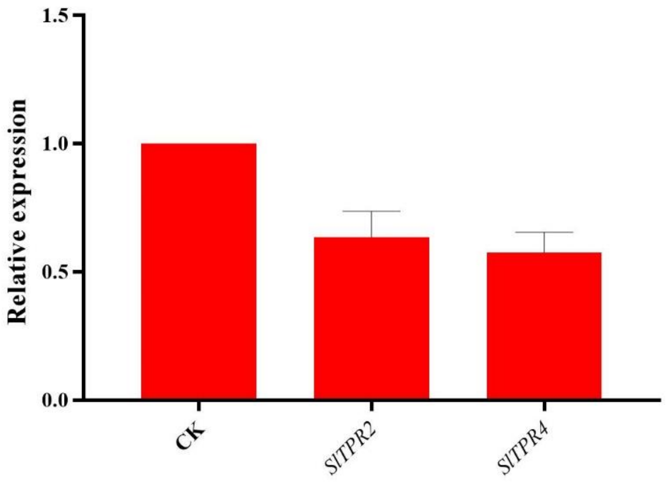

Figure S4. *SlTPR2* and *SlTPR4* gene expression in silenced plants. CK was the wild type plant in the control group.
